# Niche partitioning among dead wood-dependent beetles

**DOI:** 10.1038/s41598-021-94396-x

**Published:** 2021-07-26

**Authors:** Jakub Horák

**Affiliations:** Faculty of Forestry and Wood Sciences, Czech University of Life Sciences Prague, Kamýcká 129, 165 00 Prague, Czech Republic

**Keywords:** Ecology, Community ecology

## Abstract

Niche partitioning among species with virtually the same requirements is a fundamental concept in ecology. Nevertheless, some authors suggest that niches have little involvement in structuring communities. This study was done in the Pardubice Region (Czech Republic) on saproxylic beetles with morphologically similar larvae and very specific requirements, which are related to their obligatory dependence on dead wood material: *Cucujus cinnaberinus, Pyrochroa coccinea,* and *Schizotus pectinicornis*. This work was performed on 232 dead wood pieces at the landscape scale over six years. Based on the factors studied, the relationships among these species indicated that their co-occurrence based on species presence and absence was low, which indicated niche partitioning. However, based on analyses of habitat requirements and species composition using observed species abundances, there was no strong evidence for niche partitioning at either studied habitat levels, the tree and the microhabitat. The most likely reasons for the lack of strong niche partitioning were that dead wood is a rich resource and co-occurrence of saproxylic community was not driven by resource competition. This might be consistent with the theory that biodiversity could be controlled by the neutral drift of species abundance. Nevertheless, niche partitioning could be ongoing, meaning that the expanding *C. cinnaberinus* may have an advantage over the pyrochroids and could dominate in the long term.

## Introduction

The biotic interactions and habitat requirements in terms of abiotic conditions of a species characterize its position in the ecosystem—i.e., its ecological niche^1^. One of the most important questions in ecology is how species interact and how their niches overlap^2^. Nevertheless, the unified neutral theory of biodiversity^3^ presented an alternative to traditional niche theory^4^. It suggests that biodiversity is mainly controlled by the neutral drift of species abundances, therefore, niches and their partitioning are not particularly important in structuring communities^5^.

Many species have relatively similar habitat requirements and, furthermore, occur in the same place^1^. This has been studied fairly thoroughly for large to small^6,7^ vertebrates. There is a high probability that the smaller the organism is, the smaller and more delineated its habitat will be^8^ and the stronger its interactions (e.g., competition) could be^9^. Therefore, it is important to understand the mechanism even for small, ephemeral and delineated resources, such as tree microhabitats^10,11^. Many questions arise regarding this topic, e.g., how species share natural resources of microhabitats; which species is more successful; and how species share resources if they have the same demands^11–13^.

Highly diversified environments such as forests and other tree-dominated ecosystems offer highly diversified habitats that can be exploited by living organisms as their niches. Forests are also thought to be the most biodiverse environments in the world. The combination of soil and woody vegetation has enabled forests to serve as local biodiversity hotspots^14^. One of the unique guilds dependent on trees is that of saproxylics, i.e., the biota that is dependent on dead woody material^15^.

A relatively large number of organisms exploit the subcortical environment. Of the organisms that depend on the under-bark substrate for successful breeding, bark beetles (Scolytinae) are the best known. One of the most important reasons is their potential to become pests^16^. Nevertheless, many species of beetles that are not pests also live in the narrow space between the bark and wood^17^. The relationships within this subcortical community are still not well known^18^. Saproxylic beetles are an important ecological guild^19^. However, our knowledge of their biotic interactions and larval niches within the saproxylic community is still highly limited^17,18^.

Entomologists studying beetles mainly focus their research on adults^20^. One of the reasons for this focus is highly objective—the evaluation of dispersal and reproduction. Other reasons are rather subjective, as adults are, in most cases, much more beautiful and easier to identify than larvae. The focus on adults is also strongly related to the fact that many beetle species do not have described larvae. Nevertheless, studies on larvae are becoming more frequent^21^, and larval requirements reveal important information about the suitability of their environments^22,23^. The main activities of larvae are eating, growing, and surviving to pupation. Therefore, the information about larval dietary preferences, successful growth and environments is important^13,17,20–22^, especially that developmental stages live much longer than adults^17^.

**Figure 1:**
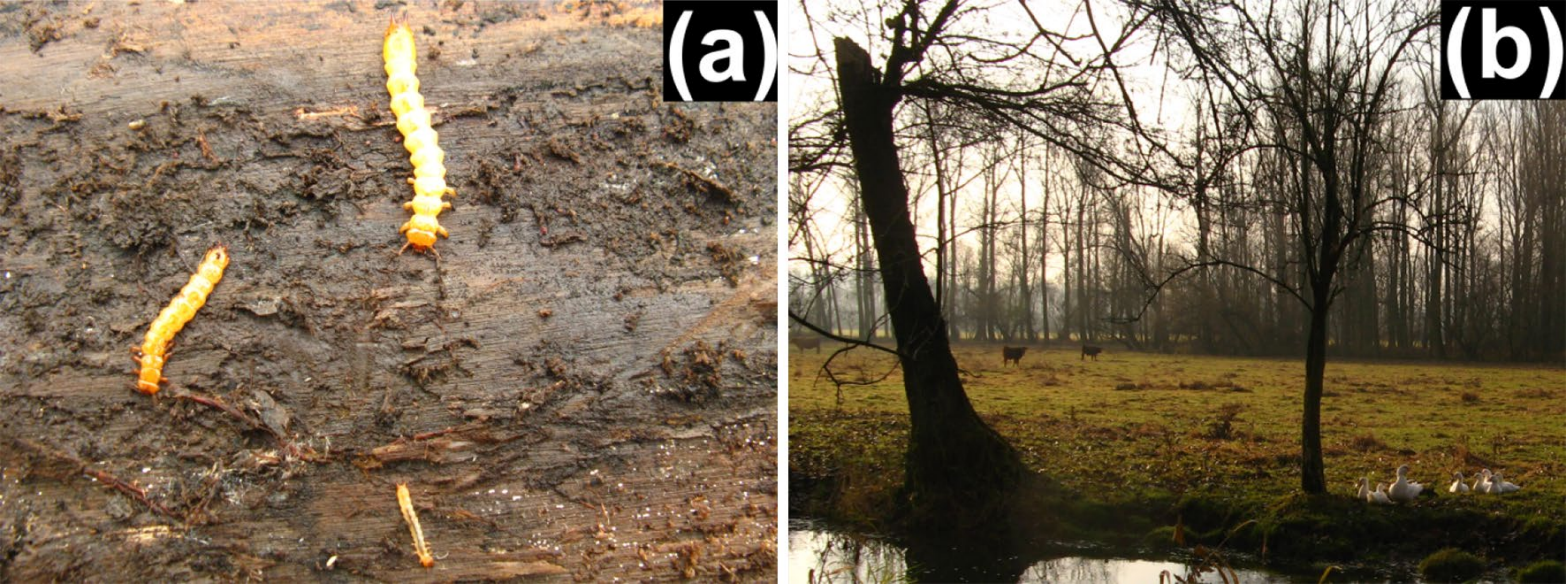
Study species and their habitat. (**a**) Illustration of the cooccurrence of all three studied species, with a larva of *Cucujus cinnaberinus* on the left, one of *Pyrochroa coccinea* at the top and one of *Schizotus pectinicornis* at the bottom. (**b**) View of the habitat where all three species cooccurred in the Czech Republic.

The main aim of this study was to find possible interspecific interactions (i.e., co-occurrences) among three obligate saproxylic species and possible partitioning of their niches in terms of their habitat requirements at the tree and microhabitat levels. The study focused on larvae of morphologically similar species with apparently similar habitat requirements, namely, one flat bark beetle (Cucujidae), *Cucujus cinnaberinus* (Scopoli), and two fire-colored beetles (Pyrochroidae), *Pyrochroa coccinea* (Linnaeus) and *Schizotus pectinicornis* (Linnaeus). All species have relatively long and flat-bodied larvae, with an abdomen bearing urogomphi, which enable them to crawl in narrow spaces between the wood and bark (Fig. 1). The species have been reported to prefer wood infested with fungi, with occasional feeding on subcortical arthropods^17^. Adults of the fire-colored beetles are mainly observed on vegetation within forested landscapes^24^. Adults of the flat bark beetle spend most of their life under or on the bark of trees^25^.

The main focus of this research was the biotic interactions and requirements of the three saproxylic beetles at the microhabitat and tree levels. Using gradual steps, I aimed to test for possible niche partitioning^26^. Specifically, I was interested in (1) biotic interactions:

1. Spatial structure of species—with the prediction of significant positive spatial autocorrelation for the studied species, i.e., clustered spatial distributions of individual species. This can indicate species avoidance in space.
2. Relative density of the species—with the prediction of differences in the observed abundances of individual species. This can indicate the predominance of some species.
3. Overlap in species occurrences—with the prediction that the species would not share habitats. This can indicate species avoidance within habitats.

I also focused on (2) species requirements:

1. Species requirements at the tree level—with the prediction that the species would prefer different tree-level parameters.
2. Species requirements at the microhabitat level—with the prediction that the most suitable microhabitat-level parameters would differ among species. These two points can indicate differences in species' habitat requirements.

And finally, I focused on (3):

1. Species composition within the environment—with the prediction that the composition would be highly differentiated. This can indicate differences in species' interactions with the environment.

## Results

By inspecting dead wood, 198 larvae of *C. cinnaberinus* (mean ± SE = 0.85 ± 0.15; min–max = 0–20 per piece of dead wood), 77 larvae of *P. coccinea* (0.33 ± 0.09; 0–12) and 72 larvae of *S. pectinicornis* (0.31 ± 0.08; 0–9) were found.

### Species interactions

**Figure 2:**
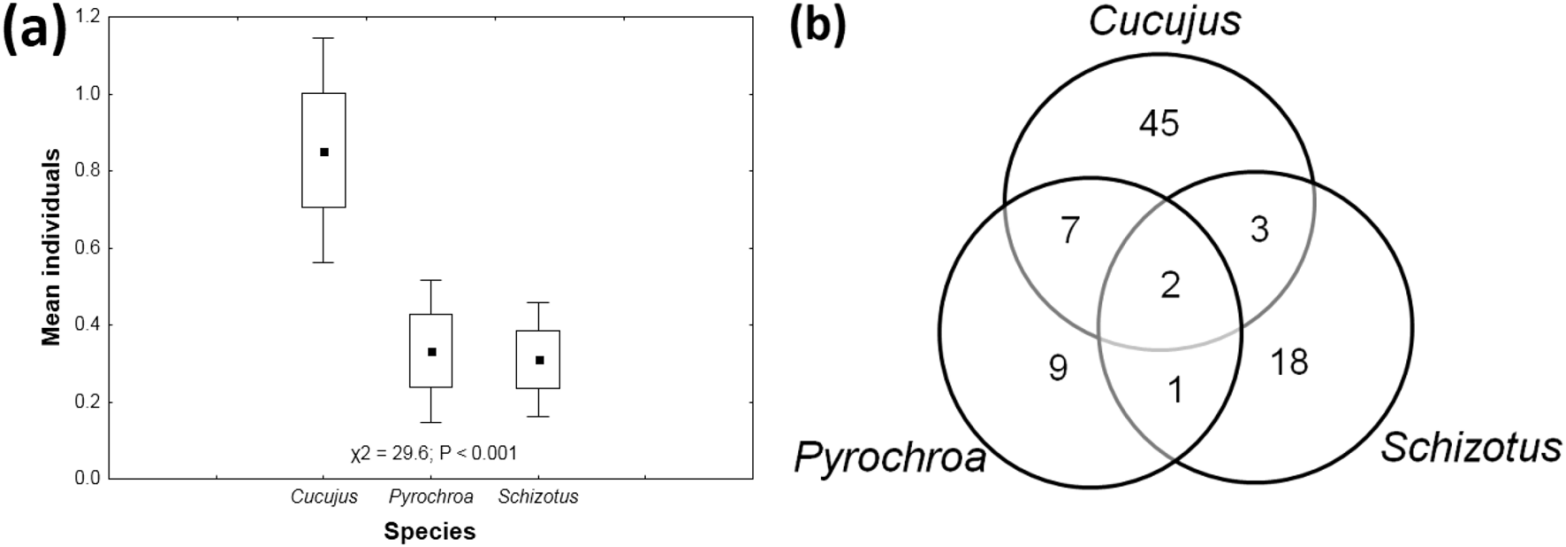
(**a**) Comparison of larval abundances among the three studied saproxylic beetles in the Czech Republic. Squares are the means; boxes are the means ± SEs; and whiskers are the means ± 1.96 SEs. (**b**) Venn diagram illustrating the numbers of observed occurrences and cooccurrences of the studied species.

Data on the abundance of individual species did not show spatial clustering (*C. cinnaberinus*: *P* = 0.241; *P. coccinea*: *P* = 0.146; and *S. pectinicornis*: *P* = 0.106). The abundance of larvae of *C. cinnaberinus* was significantly higher than that of the other two species. All possible species combinations were observed (Fig. 2). Based on the occurrences of individual species, the high C-score indicated low cooccurrences among species (Fig. S1). This indicated a segregated matrix of species occurrence.

### Species and the tree level

Based on the explained variance, the position of dead wood explained the most variance in *C. cinnaberinus* and *S. pectinicornis* abundance. This was followed by poplar as a host tree for *C. cinnaberinus*, and sun exposure for *P. coccinea*. Other predictors had only marginal effects on the abundance of the studied species at the tree level (Table S1).

Fallen dead wood pieces significantly positively influenced the abundances of *C. cinnaberinus* and *S. pectinicornis* larvae against standing dead wood. Poplar as a host tree was significantly positively related with the abundance of *C. cinnaberinus* in contrast to other tree species and increasing sun exposure had an almost negative effect on *P. coccinea* (Table 1).

**Table 1:** Interactions of larvae of the three studied saproxylic beetles with the environment at the tree level in the Pardubice Region (Czech Republic).

Species	Predictor	Estimate	t	*P*
*Cucujus cinnaberinus*	Position (lying)	− 1.246	− 2.887	**0.004**
	Poplar	0.680	1.835	0.068
	Diameter	0.005	0.718	0.473
	Bark cover	− 0.004	− 0.601	0.549
	Fungi	− 0.015	− 0.013	0.989
	Sun exposure	0.012	0.352	0.725
*Pyrochroa coccinea*	Position (lying)	− 0.852	− 1.219	0.224
	Poplar	0.353	0.633	0.527
	Diameter	0.014	1.433	0.153
	Bark cover	− 0.002	− 0.209	0.835
	Fungi	0.079	0.107	0.915
	Sun exposure	− 0.636	− 1.895	0.059
*Schizotus pectinicornis*	Position (lying)	− 1.955	− 2.687	**0.008**
	Poplar	− 0.229	− 0.395	0.693
	Diameter	− 0.012	− 0.971	0.332
	Bark cover	0.019	1.518	0.130
	Fungi	0.134	0.367	0.714
	Sun exposure	0.469	1.308	0.192

GLM was used and significant *P* values are shown in bold.

### Species and the microhabitat

The wetness of the under-bark substrate played the most important role in determining *C. cinnaberinus* and *S. pectinicornis* larval abundance. The third species, *P. coccinea*, was unaffected by the microhabitat predictors (Table S2).

A higher humidity of the under-bark substrate positively influenced *C. cinnaberinus*. This relationship was also nearly positive for *S. pectinicornis*. The other factors did not play a significant role (Table 2).

**Table 2:** Interactions of larvae of the three studied saproxylic beetles with the environment at the microhabitat level in the Pardubice Region (Czech Republic).

Species	Predictor	Estimate	t	P
*Cucujus cinnaberinus*	Bark peeling	− 0.254	− 0.996	0.320
	Wetness	0.887	3.376	**0.001**
	Bast consistency	0.187	0.678	0.498
	Mycelia	− 0.552	− 1.356	0.177
*Pyrochroa coccinea*	Bark peeling	− 0.219	− 0.542	0.588
	Wetness	0.417	1.245	0.215
	Bast consistency	0.597	1.140	0.256
	Mycelia	− 1.036	− 1.432	0.154
*Schizotus pectinicornis*	Bark peeling	− 0.243	− 0.598	0.550
	Wetness	0.732	1.873	0.062
	Bast consistency	− 0.103	− 0.259	0.796
	Mycelia	0.325	0.595	0.553

GLM was used and significant *P* values are shown in bold.

### Species composition and the environment

**Figure 3:**
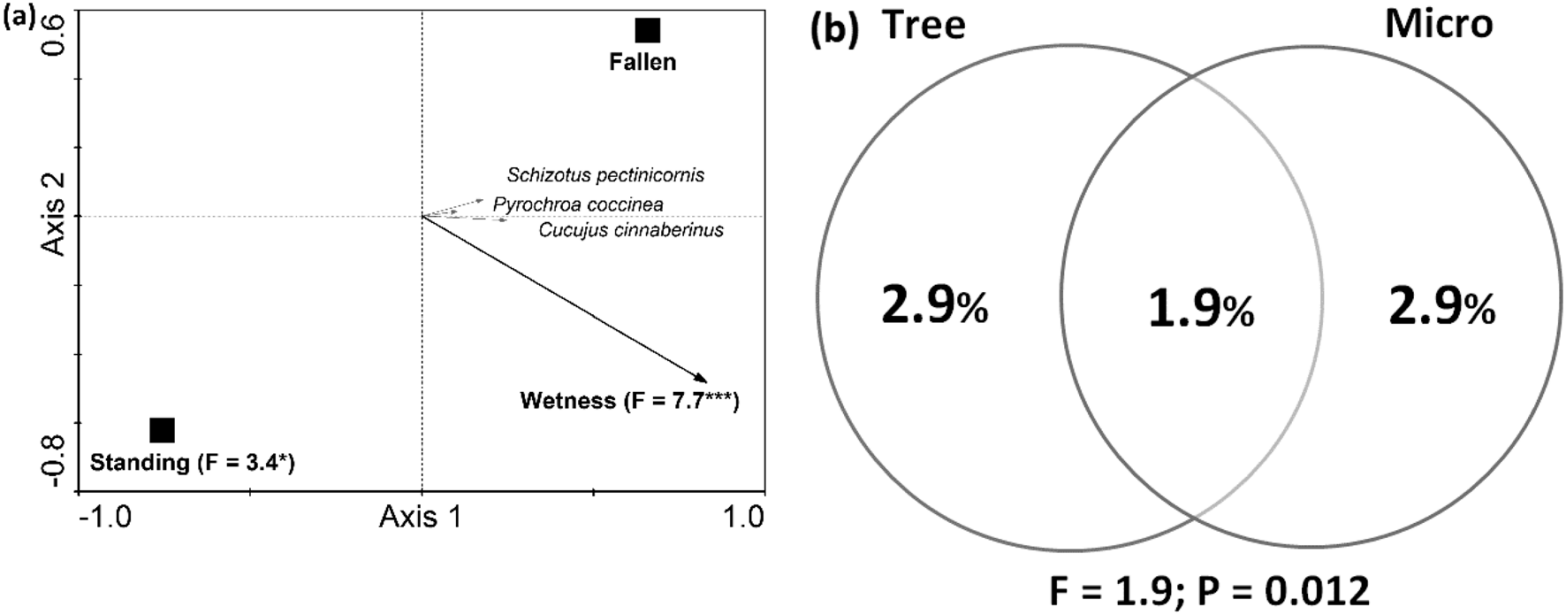
Responses of larvae of the three studied saproxylic beetles to the environment at the tree and microhabitat levels in the Czech Republic. (**a**) Only significant predictors are presented in the RDA plot; nonsignificant predictors were treated as covariables. *P < 0.05 and ***P < 0.001. (**b**) Venn diagram illustrating the partitioning of variance between the tree and microhabitat predictors.

The species composition analysis confirmed significant responses of all three species to the environment. All species were mainly associated with fallen dead wood and the gradient of wetness, with both variables mainly distributed along the first axis (R^2^ = 6.4%; F = 15.2; *P* = 0.006). Tree- and microhabitat-level predictors explained the same percentage of variance. The shared variance was lower than the independent variance (Fig. 3).

## Discussion

### Co-occurrences among the target species

The results revealed that species overlap in terms of occurrence was very low and the species mainly occurred alone. The results for the fire-colored beetles were partially consistent with the knowledge that niches of closely related species tend to differ at least in some aspects, as interspecific competition increases partitioning^1^. However, the findings also partly support the theory that biodiversity could be controlled by the neutral drift of species abundance^3^. Regarding species interactions based on their abundances, *C. cinnaberinus* was the most abundant beetle. Furthermore, this species also exhibited the largest number of larvae per sample. Large numbers of larvae in one place were also observed for the other two studied species. These results indicate occasional gregarious behaviors of these species. Such behaviors are known to occur in other saproxylic beetles, which exploit similar microhabitats^27^. However, this pattern cannot be taken as an indication of social behavior, such as that observed, e.g., in saproxylic ambrosia beetles^28^. None of the study species are not known to be significantly limited in their dispersal e.g.,^29^, as the poor dispersal might result in aggregated occurrence patterns^30^. Concerning larval aggregation, additional information that is lacking and that could help clarify the relationships among saproxylic beetles is whether larvae in one log are the descendants of a single female. There is evidence that at least in the case of *C. cinnaberinus*, more than two parents are responsible for breeding^31^. Therefore, the observed gregarious pattern is probably not only due to the fact that the larvae are related, which is known to occur in insects^32^.

### Species and their observed niche requirements

Regarding the microhabitat requirements, *C. cinnaberinus* preferred subcortical environments with a high humidity decaying bast (phloem). This result probably reflects the preference of this species for bast decayed by yeasts^17^ and dead arthropods^33^. Nevertheless, the most likely competitor of *C. cinnaberinus*, namely, *P. coccinea* (with larvae that can reach more than 3 cm in size), was rather not choosy regarding microhabitat preferences. Only *S. pectinicornis* exhibited a relationship with humid microhabitats.

In the cases of *C. cinnaberinus* and *S. pectinicornis*, there was an indication of possible niche sharing, in which they both preferred fallen dead wood. A preference for fallen trunks is consistent with the preference of these species for humid microhabitats, as logs are almost always moister than standing dead wood. However, only three cooccurrences of *C. cinnaberinus* and *S. pectinicornis* are not sufficient to support this statement. The significantly lower number of *S. pectinicornis*, its low cooccurrence with *C. cinnaberinus,* and the similar preferences for the environment lead to the hypothesis that *C. cinnaberinus* is a successful competitor. This species appears to be undergoing range expansion at the present time^34,35^ and may occupy niches that were formerly inhabited by the fire-colored beetles.

These findings indicate that *C. cinnaberinus* has a possible advantage over pyrochroids and could become dominant in the long term. More likely, it would affect the abundances of inferior competitors (i.e., pyrochroids). Nevertheless, it can also be predicted that this might be a result of the process that would lead in the future to the competitive exclusion principle to structure saproxylic communities^36,37^. Namely, this process would lead to either a behavioral or evolutionary shift toward a different ecological niche in pyrochroids. This may probably explain why *P. coccinea* was not choosy in habitat requirements. Another possibility is that *C. cinnaberinus* has stricter ecological requirements^38^ than *P. coccinea*.

The studied species did not exhibit a clear preference for categories of tree species. There were no preferences for the diameter, bark coverage, or the presence of fungi either, which confirmed the findings of previous studies on *C. cinnaberinus*^35,38^. The lack of response to the wood-inhabiting fungi could reflect the lack of response to the consistency of wood and the presence of mycelia. However, the lack of response to the peeling of the bark contrasts with the findings of previous research on *C. cinnaberinus*^38^. The most likely reason for this discrepancy is the exclusion of adults from this study.

### Sharing, partitioning, or a neutral relationship?

The result of this study appears to be consistent with the knowledge that community structure may not be driven by resource competition^39^, mainly because dead wood appears to be a rich resource (nutrients and microhabitats)^14,19^. Important features of the ecological niche include the habitat requirements and functional role of the species^1^. Therefore, one indication of niche partitioning is that one species mainly avoids the others even if all these species have highly similar dietary preferences^17^. Another possible explanation for the low niche partitioning among species is that the most abundant species, *C. cinnaberinus*, displays many signs of antipredator strategies, together with antimicrobial and antifungal functions^40^. The same adaptations could also be expected for the pyrochroids^41–43^. The most likely reason for the lack of strong niche partitioning could be at least partial consistency with neutral theory. Nevertheless, niche partitioning could be ongoing due to range expansion of *C. cinnaberinus* which could dominate in the long term.

## Methods

### Target species

The larvae of three obligately saproxylic species were studied. The first two species are in the same family of fire-colored beetles (Pyrochroidae): *P. coccinea* and *S. pectinicornis*. The third species is in the family of flat bark beetles (Cucujidae): *C. cinnaberinus*. The observed length of the larvae was 10–24 mm in *C. cinnaberinus*, 10–39 mm in *P. coccinea* and 9–26 mm in *S. pectinicornis*. The larval development of the three studied species lasts for more than 1 year ^24,25^.

All the species were identified in the field. Only very small larvae (< 10 mm) of *P. coccinea* that can be misidentified with the rare species *P. serraticonins* (Scopoli) were taken into the laboratory for detailed identification.

### Study area

The study area was situated in the Pardubice Region (Czech Republic) and totaled more than 1000 km^2^. The area has a lowland landscape dominated by crop fields, followed by grasslands and forests. This area is relatively densely populated. The elevation is between 200 and 370 m a.s.l. The climate has an annual mean temperature between 7.5 and 9 °C and precipitation between 550 and 700 mm.

During six consecutive years (from 2006 to 2012), 232 pieces of dead wood were inspected for the presence or absence of *C. cinnaberinus*, *P. coccinea*, and *S. pectinicornis*. The samples were collected throughout the year. Sites with dead wood were heterogeneous. The inspected habitat types included riparian corridors, abandoned lignicultures, commercial and conservation forests, as well as tree windbreaks and avenues. The method of bark strip removal was used^23,35,38^ to search for the larvae of the studied species. Nearly every piece of the dead wood found (limbs, logs, stumps, snags, fallen trees, etc.) was studied. As there could be substantial variation in the carrying capacity of such dead wood, I used equal-stratified sampling^37^ with the standardization of one sample of the same size per dead wood piece^44^.

### Environmental parameters

Samples were taken from each piece of dead wood. Specifically, one bark strip (≈ 0.1 m^2^) was peeled away and replaced after collecting the data on the larvae and environment.

Based on the previous research^38^, data on the studied environment were divided into two hierarchical levels that reflected the demands of saproxylic beetles living under the bark. The first, tree level included independent variables that can be observed without modifying the dead wood. Specifically, the tree species (or genus) was identified, but due to a large number of tree species and statistical parsimony^45^, only the poplar tree (*Populus* spp.; N = 127), other broadleaved trees (N = 89) and conifer tree (N = 16) categories were used. Other broad-leaved trees included oak (*Quercus*), ash (*Fraxinus*), alder (*Alnus*), cherry (*Prunus*), willow (*Salix*), maple (*Acer*), horse chestnut (*Aesculus*), birch (*Betula*), hornbeam (*Carpinus*), beech (*Fagus*), locust (*Robinia*), rowan (*Sorbus*) and linden (*Tilia*); conifer trees were represented by spruce (*Picea*) and pine (*Pinus*). The studied species preferred medium stage decayed dead wood. An insignificant effect of tree chemistry^35^ compared to the attraction effect of living trees on insects can be expected^13^. The second variable, the position of dead wood, was designated as either standing (N = 108) or fallen (N = 124). The diameter of the sampled dead wood was the third variable (mean ± SE = 42.3 ± 1.5 cm; min = 2.0 cm, max = 149.0 cm). The fourth variable was the bark cover of the dead wood piece (79.4 ± 1.5%; 10.0–100.0%). The presence (N = 74) or absence (N = 158) of fruiting bodies of wood-inhabiting fungi was noted as a fifth variable. The level of sun exposure was estimated as the last variable at this level, with the following three categories: shaded (N = 59), semi-shaded (N = 67) and sun exposed (N = 106), following the previously published methodology^38^.

The second, microhabitat level of sampling included characteristics found under the bark. The stage of bark peeling, scored as good (N = 134), medium (N = 51) and poor (N = 47), was measured based on the power applied by a screwdriver needed to peel the bark. The wetness of the under-bark substrate was estimated as wet with the majority of the sample covered by humidity (N = 106); partly wet (N = 58) or dry, with no observed humidity (N = 68). The consistency of the bast (phloem) was reflected by its color as follows: yellow (N = 38) when fresh, brown (N = 36) when decayed and black when crumbled (N = 258). Finally, the presence (N = 75) or absence (N = 157) of fungal mycelia was noted.

### Statistical analyses

The effect of spatial autocorrelation of the studied species was tested using Moran’s I in SAM v4.0.

Differences in the abundances among individual species within samples were computed using Friedman’s ANOVA. This analysis was performed using Statistica 13.2.

The co-occurrence analysis was performed in R 3.0.2 using the EcoSim R package^46^ to analyze possible aggregation of species occurrence. Occurrences were randomized, and species and site data were fixed in the null model. The number of burn-in iterations was 500. Comparison of simulated and observed matrices was used to confirm the proper use of the randomization algorithm^46^.

Regarding the analyses of relationships with the environment, there was high multicollinearity for tree species; thus, only a predictor specifying whether the host tree was a poplar (55%) was used in the final analysis. This analysis was performed using the HH package^47^. All independent predictors were analyzed as continuous variables; i.e., categorical ones were transformed to a semi-quantitative scale. The variance explained by a particular independent variable was computed separately for the tree and microhabitat levels using hierarchical partitioning in the hier.part package^48^. The relationship between larval abundance and the environment was computed using two generalized linear models (tree and microhabitat). These analyses were performed in R 3.0.2.

The influence of the studied environment on the species composition of the studied species was computed using redundancy analysis (RDA) with 9999 permutations. Variance partitioning among tree- and microhabitat-level predictors was also performed. An inflation factor was used to reflect potential multicollinearity among independent variables, which was not observed. These analyses were performed in CANOCO 4.5. The results for the species composition were visualized in CanoDraw 4.12; species and significant predictors were visualized, while other predictors were included as covariables.

## Supplementary Information

Supplementary Figure S1.

Supplementary Tables.

## Data Availability

Data are available upon request.
