# Supplementary material for: Niche partitioning among dead wood-dependent beetles
Source: Sci Rep. 2021 Jul 26;11:15178. doi: 10.1038/s41598-021-94396-x (PMC8313673; doi:10.1038/s41598-021-94396-x)
Supplement: Supplementary file 1 — Supplementary Figure S1. [file 41598_2021_94396_MOESM1_ESM.docx]

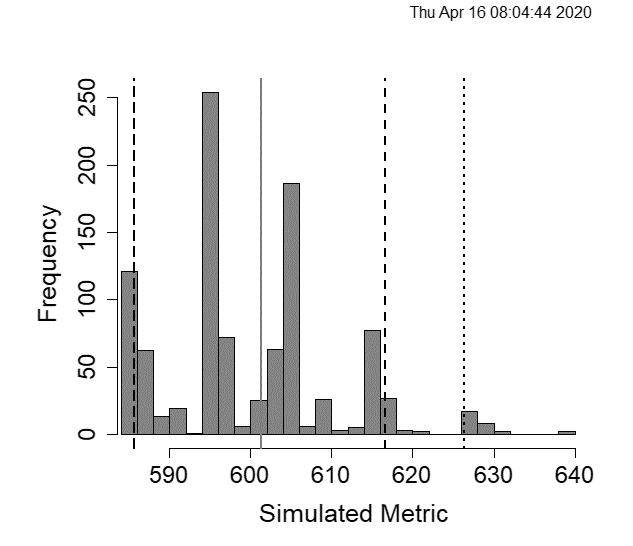


**Fig. S1**

**Fig. S1.** C-score histogram of simulated metric values (bars) among the three studied saproxylic beetles in the Czech Republic. The vertical gray line indicates the computed metric for the data; two vertical long-dash lines indicate the 95% one-tailed cutpoints; and the short-dash line indicates the 95% two-tailed cutpoint. Occurrences were randomized, and species and site data were fixed in the null model. The number of burn-in iterations was 500.
