# Supplementary material for: Niche partitioning among dead wood-dependent beetles
Source: Sci Rep. 2021 Jul 26;11:15178. doi: 10.1038/s41598-021-94396-x (PMC8313673; doi:10.1038/s41598-021-94396-x)
Supplement: Supplementary file 2 — Supplementary Tables. [file 41598_2021_94396_MOESM2_ESM.docx]

**Supplementary Tables**

**Supplementary Table S1.** Percentage of variance explained by tree-level variables for the three studied saproxylic beetles in the Pardubice Region (Czech Republic).

|  | ***Cucujus cinnaberinus*** | | ***Pyrochroa coccinea*** | | ***Schizotus pectinicornis*** | |
| --- | --- | --- | --- | --- | --- | --- |
|  | **Independent** | **Joint** | **Independent** | **Joint** | **Independent** | **Joint** |
| Position | 3.60 | 0.35 | 0.45 | -0.23 | 3.35 | 0.66 |
| Poplar | 1.57 | 0.27 | 0.07 | -0.07 | 0.05 | 0.02 |
| Diameter | 0.09 | -0.07 | 0.53 | -0.15 | 0.87 | 0.49 |
| Bark cover | 0.19 | 0.10 | 0.00 | 0.00 | 0.58 | -0.17 |
| Fungi | 0.07 | -0.05 | 0.02 | 0.01 | 0.03 | -0.03 |
| Sun exposure | 0.14 | 0.20 | 1.18 | -0.17 | 0.90 | 0.23 |

**Supplementary Table S2.** Amount of variance explained by microhabitat-level variables for the three studied saproxylic beetles in the Pardubice Region (Czech Republic).

|  | ***Cucujus cinnaberinus*** | | ***Pyrochroa coccinea*** | | ***Schizotus pectinicornis*** | |
| --- | --- | --- | --- | --- | --- | --- |
|  | **Independent** | **Joint** | **Independent** | **Joint** | **Independent** | **Joint** |
| Bark peeling | 0.48% | -0.21% | 0.24% | 0.00% | 0.13% | -0.07% |
| Wetness | 5.08% | -0.33% | 0.58% | -0.12% | 1.87% | -0.08% |
| Consistency | 0.28% | -0.08% | 0.55% | 0.02% | 0.03% | -0.01% |
| Mycelia | 0.84% | -0.04% | 0.77% | -0.09% | 0.15% | -0.01% |
